# Symptomatic, Alexithymic, and Suicidality-Related Features in Groups of Adolescent Self-Harmers: A Case-Control Study

**DOI:** 10.3390/ejihpe13050067

**Published:** 2023-05-19

**Authors:** Alessia Raffagnato, Marina Miscioscia, Rachele Fasolato, Sara Iannattone, Perla Valentini, Eleonora Sale, Michela Gatta

**Affiliations:** 1 Department of Woman and Child’s Health, Padua University Hospital, 35128 Padua, Italy; 2 Department of Developmental Psychology and Socialization, University of Padua, 35131 Padua, Italy; 3 Department of General Psychology, University of Padua, 35131 Padua, Italy; 4 Department of Communication Sciences, Humanistic and International Studies: History, Culture, Languages, Literature, Arts, Media, University of Urbino ‘Carlo Bo’, 61029 Urbino, Italy

**Keywords:** alexithymia, non-suicidal self-injury, NSSI, suicide, suicidal ideation, adolescence

## Abstract

Non-suicidal self-injury (NSSI) is an increasing phenomenon among both clinical and nonclinical adolescent groups and is associated with several psychopathological symptoms, in addition to being one of the main risk factors for suicidality. Nevertheless, differences between clinical and nonclinical samples of self-harmers in symptom dimensions, alexithymia, suicidality, and NSSI-related variables are still scarcely investigated. The current study aimed to fill this gap by enrolling a sample of Italian girls (age range: 12–19 years) that included 63 self-harmers admitted to mental health outpatient services (clinical group), 44 self-harmers without admission to mental health services (subclinical group), and 231 individuals without an NSSI history (control group). Questionnaires investigating psychopathological symptoms, alexithymia, and NSSI-related variables were administered. The results highlighted that all symptom-related variables and alexithymic traits were more severe in the NSSI groups than in the control group; in particular, self-depreciation, anxiety, psychoticism, and pathological interpersonal relationships were distinguished between the clinical and subclinical groups. Compared to the subclinical group, the clinical group was characterized by higher NSSI frequency, NSSI disclosure, self-punishment as the main reason for engagement in NSSI, and suicidal ideation. These findings were then discussed in terms of clinical practice and primary and secondary prevention in the adolescent population.

## 1. Introduction

Non-suicidal self-injury (NSSI) has been an increasing phenomenon among adolescents [1], especially during the period of the COVID-19 outbreak [2,3,4]. It occurs both in clinical (i.e., individuals who access mental health services) [5] and nonclinical (i.e., individuals involved in a nonclinical context) samples [6,7]. The lifetime prevalence of NSSI in nonclinical samples varies from 12.6% to 21% [6,7], while in clinical samples, the percentage reaches 50% [5]. With regard to psychiatric samples of adolescents, Kaess and colleagues (2013) [8] estimated that the prevalence rate for occasional NSSI (i.e., at least one, but less than five NSSI acts in the last year) was 60%, whereas that for repetitive NSSI (i.e., five or more NSSI acts in the last year) was 50%. Individuals with repetitive NSSI were found to report mental health disorders, cognitive and attentional deficits, a history of child abuse, and suicidal behavior more frequently than those without repetitive NSSI [9,10]. Consequently, high NSSI frequency has been considered a clinical severity marker [10,11].

Cassels and Wilkinson (2016) [12] described NSSI as a transdiagnostic phenomenon: in clinical and community samples, it was found to be associated with both externalizing [13,14] and internalizing disorders [12,15], in contrast with a long-term study that considered NSSI a symptom of borderline personality disorder only [16]. In other cases, NSSI could be present even without a psychiatric comorbidity, especially in community samples [12]. However, the literature revealed that NSSI is associated with specific psychopathological features, such as depressive traits [17], anxiety [15,18], somatization [19,20], interpersonal sensitivity [6,21], psychoticism [22], alexithymia [23,24], and hostility [25]. Furthermore, as specified by Valencia-Agudo and colleagues (2018) in a literature review, some of the strongest predictors of non-suicidal self-injury were depressive symptoms, as well as general psychological distress [26]. Negative social relationships and life stress events [27]—in both clinical and nonclinical samples [28]—could make the individual more vulnerable to the disruption of emotion regulation [28]. Moreover, although the NSSI act does not have a suicidal purpose, an association between NSSI and suicidal phenomena has been frequently reported [29]. A prospective longitudinal study highlighted that NSSI was a risk factor for subsequent suicidal attempts in a sample of adolescents with major depressive disorder [30]. Asarnow and colleagues (2011) [31] found that more than one-third of the sample of outpatients with NSSI had a history of suicidal attempts. Hence, NSSI represents a significant risk factor for suicidal ideation and suicidal behaviors, which are increasing among adolescents [32], even after controlling for socioeconomic and psychosocial factors [33].

Regarding NSSI functions, Rodav and colleagues (2014) [17] highlighted that, in their study, adolescents often engaged in NSSI for four main reasons: “internal emotion regulation” (such as self-punishment, to relieve feelings of sadness, to distract from unpleasant memories), “external emotion regulation” (such as to release anger or frustration), “social influence” (such as to obtain attention from others), and “sensation seeking”. Other studies found that NSSI may lead to increased positive feelings, thus fostering the reiteration of the NSSI act [34,35]. These different kinds of NSSI functions were found both in community samples [36,37] and in clinical samples [38,39]. A meta-analysis conducted by Taylor and colleagues (2018) [40] highlighted a greater prevalence of the intrapersonal NSSI function (66–81% of the subjects); only 32–56% of the subjects reported an interpersonal NSSI function. According to Gross’s (2014) model [41], NSSI is a regulator of emotions, suggesting that non-suicidal self-injurers had a deficit in emotion regulation abilities [42]. Another relevant aspect is the disclosure of NSSI acts, which is the voluntary communication to somebody about NSSI [43,44]. Peers were found to be the main recipient of an NSSI disclosure, followed by family members, mental health professionals, and teachers [43,44]. Rates of NSSI disclosure to informal sources (e.g., friends, parents) were found to be higher than those to formal sources (e.g., health professionals) [44]. Moreover, individuals who disclosed NSSI reported more severe NSSI engagement [43,45] and higher suicidal ideation [45] than those who did not. The literature highlighted a high rate of variability regarding NSSI disclosure, ranging from 17% to 89%, across studies [44]; in particular, NSSI disclosure was higher in samples of self-injurers involved in help-seeking behaviors [44]. NSSI disclosure and help-seeking behaviors have been considered as two independent variables [44] and, in general, rates of help-seeking behaviors have been found to be lower than rates of NSSI disclosure [46,47]. As a matter of fact, few individuals seek professional help prior to or after the NSSI incident, with a percentage of approximately 8–9% [48]. Other studies showed that in both adult and adolescent samples, only half of the people engaging in NSSI received psychiatric care [49,50]. Lustig and colleagues (2021) [51] found that young people sought help approximately20 months after their first NSSI act and 2 years after their first NSSI thought, without any sex difference. Moreover, they surmised that those who seek professional help later develop more severe mental health impairments over time. Wilson and Deane (2012) [52] previously described the effect of help-negation, characterized by help-avoidance and low help-seeking intentions from professionals, friends, and family. Despite the literature having widely demonstrated that NSSI is a crosswise phenomenon that arises both in clinical and nonclinical samples [26,36,38,39,53], few studies have compared clinical and nonclinical samples in terms of NSSI. Gandhi and colleagues (2021) [7], considering a clinical sample of adult patients from inpatient/outpatient psychiatric departments and a nonclinical sample of university students, found that the clinical sample presented greater damage regarding sense of self than the nonclinical sample. In any case, irrespective of the kind of sample, lifetime NSSI was associated with higher disturbed and lower consolidated identity. Conversely, Meszaros and colleagues (2020) [54], assessing the psychopathological features associated with NSSI across clinical and nonclinical groups of adolescents, highlighted that higher NSSI frequency was associated with a higher level of psychopathology, regardless of the group. To the best of our knowledge, there are no other studies that, considering the adolescent population, directly compare clinical and nonclinical groups of self-harmers on symptom and alexithymic dimensions and NSSI-related features. However, extensive research in this area is needed since the risk factors might be different for clinical and nonclinical groups [18].

The current study aimed to increase knowledge of the features that characterize young people with NSSI. Considering its severity in terms of associated psychopathologies and self-harming-related characteristics, we focused on the factors that distinguish between self-harmers admitted to mental health services (i.e., clinical group) and self-harmers who did not (i.e., subclinical group). To this end, we enrolled a sample of Italian adolescent girls that included 63 self-harmers admitted to mental health outpatient services (clinical group), 44 self-harmers without admission to mental health services (subclinical group), and 231 individuals without an NSSI history (control group).

The objectives of the study were as follows:

(1) To evaluate group differences in symptom dimensions and alexithymic features in order to pinpoint which factors may be associated with NSSI per se and mental health service utilization among non-suicidal self-injurers. We considered some of the variables previously found to be related to NSSI, namely, anxiety, somatization, interpersonal sensitivity, psychoticism, hostility, depressive traits such as negative mood, self-depreciation and pathological interpersonal relationships, and difficulty in identifying and communicating feelings (i.e., two dimensions of alexithymia). Asthe literature showed that these variables are related to NSSI per se, we expected non-suicidal self-injurers (i.e., clinical and subclinical groups) to report more severe scores on all of the abovementioned variables than individuals without a history of NSSI (i.e., control group). The association between these variables and NSSI has been found in self-harmers enrolled in both clinical and nonclinical contexts, but we are unaware of any previous study that has considered these variables and directly compared adolescent self-injurers admitted to mental health services with those who did not. In light of this, we aimed to proceed in an explorative way with regard to the comparison between the clinical and subclinical groups.

(2) To evaluate the differences in suicidality and NSSI-related variables (i.e., frequency, functions, disclosure) between the clinical and subclinical groups to demonstrate that accessing specialistic services is a sign of higher severity. For this second aim, we only considered the clinical and subclinical groups because the adolescents in the control group did not report a history of NSSI. We hypothesized a greater likelihood of finding suicidal ideation in the clinical group (i.e., subjects who attended the mental health service) compared to the subclinical group. Moreover, we expected that the clinical and subclinical groups differed in NSSI frequency, which has been considered a clinical severity marker, with a higher NSSI frequency in the clinical group. Since NSSI frequency was considered a clinical severity marker and was associated with intrapersonal functions of NSSI, we also hypothesized a higher likelihood of finding the use of intrapersonal functions (e.g., reducing negative emotions, self-punishment, and urgency and desire) in the clinical group. Lastly, we aimed to investigate whether the two groups differed in terms of NSSI disclosure.

## 2. Materials and Methods

### 2.1. Participants and Procedure

We involved a clinical group of 63 girls with NSSI accessing two types of neuropsychiatric outpatients services for adolescents: territorial and hospital (age range: 12–18 years, M = 15, SD = 1.43). In addition, a nonclinical sample of 275 girls recruited from three high schools was considered. The nonclinical sample was divided into two subgroups: the first one labeled “subclinical group” was characterized by 44 non-suicidal self-injured adolescents who declined to use mental health services (age range: 14–19 years, M = 16.2, SD = 1.65) and the second one labeled “control group” was characterized by 231 adolescents without a history of NSSI (age range: 14–19 years, M = 16.6, SD = 1.53). Regarding the clinical sample, data were acquired through questionnaires (see Section 2.2) administered to patients during outpatient clinical interviews for diagnostic assessment at neuropsychiatric units after obtaining informed consent. Patients were selected for the current study according to the presence of an NSSI history and an outpatient clinical assessment procedure. Data from the nonclinical sample were acquired in a school-based setting through the administration of the same questionnaires after obtaining informed consent and with the cooperation of teachers and school directors. For both samples, the recruitment involved obtaining the informed consent of the adolescents and their parents. Moreover, permission from school directors was required for the enrolment of the nonclinical sample. The exclusion criteria for the clinical group were as follows: diagnosis of autism spectrum disorder and intellectual disability, current inpatient hospital admission, and the absence of a history of NSSI over the last year. The exclusion criterion for the nonclinical group was the absence of informed consent from the subject and her parents. In the clinical group, the history of NSSI was evaluated during clinical interviews for diagnostic assessment, while in the nonclinical group, the history of NSSI was examined through a dichotomous yes/no question about self-harming (“Have you ever self-injured during the last year?”). Other NSSI-related features (see Section 2.2.1) were only investigated in subjects who reported a history of NSSI. Table 1 describes the categories of people with whom non-suicidal self-injurers had discussed NSSI. The study was part of a larger one and it was conducted according to the Declaration of Helsinki and approved by the local ethics committee (CESU, October 2019, prot.23).

### 2.2. Tools

The following questionnaires were chosen according to the psychodiagnostic procedure regularly followed by clinicians during clinical interviews for diagnostic assessment at neuropsychiatric units. Moreover, this set of questionnaires enabled the evaluation of specific variables that have been considered relevant to NSSI (e.g., NSSI related-features and functions, alexithymia, clinical symptoms).

#### 2.2.1. Non-Suicidal Self-Injury Questionnaire

The NSSI questionnaire is an ad hoc instrument that investigates NSSI-related features [55]. The items of the questionnaire were created on the basis of the DSM-5 criteria of NSSI [56]. Specifically, it includes questions relating to the presence of an NSSI event that occurred in the last year; NSSI frequency, classified as repetitive (more than five NSSI acts in the last year) or occasional (one to four acts in the last year); NSSI disclosure and the categories of people self-injurers have talked with; and functions of NSSI. The questionnaire considers the following NSSI functions: reducing negative emotions, interpersonal difficulties management, self-punishment, and urgency and desire (intended as positive sensation-seeking). Each function was evaluated on a three-point Likert scale, ranging from 1 (disagree) to 3 (completely agree).

#### 2.2.2. Symptom Checklist 90–R (SCL-90-R) ([57]; Italian Version: [58])

The revised version of Symptom Checklist-90 (SCL-90-R) is a standardized self-report questionnaire evaluating clinical symptoms in the last week. It is composed of 90 items, with each being evaluated on a five-point Likert scale. It includes nine symptom scales: somatization, obsessive–compulsive disorder, interpersonal sensitivity, depression, anxiety, hostility, phobic anxiety, paranoid ideation, and psychoticism. The sum of the scores for each item is the Global Score, which is considered an index of global clinical dysfunction. In line with previous literature concerning NSSI, we considered the somatization, interpersonal sensitivity, anxiety, hostility, and psychoticism scales. Regarding the internal consistency of the instrument, Prunas and colleagues (2012) [59] found Cronbach’s alpha values ranging from 0.70 to 0.96.

#### 2.2.3. Children’s Depression Inventory (CDI) ([60,61]; Italian Version: [62])

This is a self-report standardized questionnaire used to assess depressive symptoms in children and adolescents. It is composed of 27 items, in which subjects are asked to respond according to their mood in the previous two weeks. For each item, the respondent chooses one of three sentences that best describes their feelings, and each answer is evaluated from 1 to 3. The total score, which measures the severity level of depression, is obtained by summing all scores. The instrument includes five scales indicating different depressive traits: pathological mood, self-depreciation, ineffectiveness, anhedonia, and pathological interpersonal relationships. In line with the objectives of our study, we only considered the pathological mood, self-depreciation, and pathological interpersonal relationships scales. Moreover, we considered item 9 separately in order to assess suicidality. This item is characterized by three statements: “I do not think to kill myself”, “I think to kill myself but I would not do it”, and “I want to kill myself”. Pertaining to psychometric properties, Frigerio and colleagues (2001) [63] found an adequate internal consistency of the instrument (Cronbach’s alpha value = 0.80).

#### 2.2.4. Toronto Alexithymia Scale (TAS-20)

The Toronto Alexithymia Scale-20 (TAS-20) ([64]; Italian version: [65]) is a 20-item self-report questionnaire that evaluates three factors defining alexithymia: difficulty in identifying feelings (DIF), difficulty in describing feelings (DDF), and externally oriented thinking (EOT). A total score is obtained by summing the scores of all three factors. On the basis of the literature concerning NSSI, in our study, we only considered the DIF and DFF scales. The validity of this instrument in the pediatric population has been demonstrated by different studies (e.g., [66,67,68]). The Italian version of the TAS-20 has good internal reliability (Cronbach’s alpha values: 0.52–0.75 for the general population and 0.54–0.82 for clinical samples) [65].

### 2.3. Data Analysis

To analyze the differences between the three groups (i.e., clinical, subclinical, and control) in the alexithymic traits and symptom-related variables of interest, a multivariate analysis of covariance (MANCOVA) controlling for the subjects’ age was run. The dependent variables were the scales for somatization, interpersonal sensitivity, anxiety, hostility, and psychoticism on the SCL-90-R, TAS-20 DIF, and TAS-20 DDF scales and the CDI pathological mood, pathological self-depreciation, and pathological interpersonal relationships scales. These variables were selected because they were considered the most relevant based on the literature. Moreover, we were interested in exploring which specific dimensions of depression and alexithymia could be discriminated among the three groups. In the case of a significant main effect, pairwise comparisons across groups were conducted using Bonferroni-corrected post hoc tests.

Subsequently, considering the subclinical and clinical groups only, chi-square (χ^2^) tests for categorical variables were employed to examine the association between the group to which the participants belonged and specific variables linked to suicidality, namely, frequency in the last year (two levels: between 1 and 4 vs. more than 5), NSSI disclosure (two levels: yes vs. no), and different functions of NSSI acts (i.e., reducing negative emotions, interpersonal difficulties management, urgency and desire, and self-punishment; three levels each: 1, 2, 3), and the presence of suicidal ideation; this last aspect was investigated by means of item 9 of the CDI (three levels: “I do not think to kill myself”, “I think to kill myself but I would not do it”, and “I want to kill myself”).

For all analyses, the Jamovi statistical software version 2.3.17 [69] was used and a two-tailed level of significance of *p* < 0.05 was considered.

## 3. Results

### 3.1. Symptom Dimensions and Alexithymic Traits: Comparison between Clinical, Subclinical, and Control Groups

The results of the MANCOVA revealed an overall significant effect of group membership (Wilk’s λ = 0.617, F (20, 584) = 7.97, *p* < 0.001), with age as a significant covariate at the multivariate level (Wilk’s λ = 0.899, F (10, 292) = 3.27, *p* < 0.001). Subsequent univariate analyses showed significant differences across the three groups on all of the symptom dimensions and alexithymia-related variables we investigated (Table 1), while the effect of age was only significant regarding the interpersonal sensitivity (F (1, 301) = 4.07, *p* = 0.044) and hostility (F (1, 301) = 4.17, *p* = 0.042) scales of the SCL-90-R, the DIF scale of the TAS-20 (F (1, 301) = 5.54, *p* = 0.019), and the pathological self-depreciation scale of the CDI (F (1, 301) = 8.48, *p* = 0.004).

The subsequent Bonferroni-corrected post hoc tests showed that, on the SCL-90-R anxiety and psychoticism scales and on the CDI pathological self-depreciation scale, the clinical group obtained significantly higher scores than the other groups and, in turn, the subclinical group scored higher than the control group (Table 2). Moreover, significant differences emerged between the clinical and control groups and between the subclinical and control groups on the SCL-90-R interpersonal sensitivity and hostility scales, TAS-20 DIF and DDF scales, and CDI pathological mood scale (Table 2). Finally, the clinical group also reported significantly higher scores than the control group for the SCL-90-R somatization scale and CDI pathological social relationship scale, but only on the latter scale was there a statistically significant difference between the clinical and subclinical groups (Table 2).

### 3.2. Differences in Suicidality-Related Variables between Clinical and Subclinical NSSI Groups

Chi-square tests showed a significant association between the group to which the participants belonged (i.e., clinical vs. subclinical) and NSSI frequency over the last year (*χ*^2^(1, 89) = 11; *p* < 0.001). Specifically, 63% of the clinical group reported more than five NSSI acts in the last year, whereas only 27.9% of the subclinical group showed a similar NSSI frequency. A significant association also emerged regarding NSSI disclosure (*χ*^2^(1, 85) = 8.54; *p* = 0.003): 90.7% of the clinical group communicated with somebody about NSSI, whereas only 64.3% of the subclinical group reported the same behavior. Concerning different functions of NSSI acts, the chi-square tests revealed that the group to which participants belonged was only significantly associated with self-punishment (*χ*^2^(2, 99) = 9.17; *p* = 0.01); specifically, the clinical group reported engaging in NSSI as a form of self-punishment more frequently than the subclinical group (58.2% vs. 31.8%, respectively). Finally, we also found a significant association between the group to which participants belonged and suicidal ideation (i.e., item 9 of the CDI; *χ*^2^(2, 96) = 10.3; *p* = 0.006): 57.7% of the clinical group (vs. 52.3% of the subclinical group) had thought about killing themselves even though they would not do it, whereas 23.1% of the clinical group (vs. 4.5% of the subclinical group) declared that they wanted to kill themselves.

## 4. Discussion

Considering three groups (i.e., control, subclinical, and clinical), the first aim of our study was to investigate group differences in symptom dimensions and alexithymic traits in order to identify potential variables associated with NSSI and mental health service admission among adolescent non-suicidal self-injurers. We found that all alexithymic and symptom-related variables showed an increasing severity score among the three groups (i.e., from control to subclinical to clinical), but only for anxiety, psychoticism, and self-depreciation did this increment reach statistical significance in all pairwise comparisons. Our findings could be read in light of previous studies that have reported that anxiety symptoms are related to an inability to tolerate emotional distress [70], while the association between NSSI and psychotic experiences, despite being less investigated in the literature, could indicate a higher level of psychological distress and a more severe clinical picture [71]. Moreover, the psychoticism dimension of the SCL-90-R is a heterogeneous construct, encompassing different aspects of the near-psychosis experiences labeled detachment (i.e., never feeling close to another person, loneliness), metacognitive dysfunction (i.e., incorrect attribution of the ownership and agency of thoughts), self-accusation (i.e., contents of thoughts related to inadequacy, guilt, death, and punishment) [72], and interpersonal alienation [73]. Taken together, it appears that the interpersonal and cognitive dysfunctions typical of near-psychosis experiences are more pronounced when moving from the control group to the subclinical and clinical groups; therefore, they may underlie more impaired psychological functioning, which could ultimately lead to NSSI acts. In addition, the clinical group seemed to perceive a more severe general distress than the other two groups (i.e., control and subclinical), and, in turn, the subclinical group reported a more severe level of general distress than the control group. Self-depreciation, besides being considered a depressive trait [60,61], refers to an impairment in self-esteem, which is a dimension of identity according to the Alternative Model for Personality Disorders proposed in section III of the fifth version of the Diagnostic and Statistical Manual of Mental Disorders [55]. As such, it seems that the three groups differ in this identity aspect, which becomes more impaired when moving from the control group to the subclinical and then the clinical group. These findings are consistent with a previous study conducted by Gandhi and colleagues (2021) [7], who focused on the sense of self and, in line with our results, found that NSSI itself was associated with identity deficiencies, but in the clinical group, the damage was greater [7].

Subsequently, the remaining variables considered in the first aim of our study are related to intrapersonal and interpersonal dimensions. On the one hand, the variables concerning the intrapersonal dimension, which encompasses aspects referring to the individual’s inner perception and features (i.e., difficulty in identifying and communicating feelings, pathological mood, interpersonal sensitivity, and hostility), differed between the control and NSSI groups (i.e., clinical and subclinical). Therefore, these variables could be associated with the onset of NSSI itself, as already widely demonstrated in the literature [6,17,23,24,25]. On the other hand, the interpersonal dimension (i.e., pathological social relationships) seems to better differentiate the non-suicidal self-injurers belonging to the subclinical group from those belonging to the clinical group. Many studies have already suggested the importance of social relationships for general mental health [74,75,76] and the role of social competencies in NSSI onset [19], but the findings herein would indicate that difficulties in social relationships specifically characterize outpatients with NSSI. We hypothesize that individuals belonging to the subclinical group, despite having NSSI, experience more functional social relationships, which can operate as a buffer factor, preventing the deterioration of global mental health and, as a consequence, admission to mental health services. With regard to somatization, we only found a statistical difference between the clinical and control groups, while the subclinical group reported intermediate levels of severity. Thus, it seems that somatization is less able to distinguish the three groups compared to other variables. This finding is consistent with our previous studies [19,77], in which NSSI patients with somatization showed a more severe psychopathological picture compared to those without somatic symptoms. Nevertheless, future studies are required to deepen understanding of the potential differences between clinical and nonclinical samples in the association of NSSI with somatization. Moreover, a significant effect of age was found on some of the investigated variables, specifically on self-depreciation, hostility, interpersonal sensitivity, and difficulty in identifying feelings. As highlighted above, these variables refer to the identity dimension and the intrapersonal emotion dimension. Previous studies have already shown that adolescence is a critical period for the development of identity [78] and changes in emotion processing [79,80,81]. As a consequence, because the age of our total sample encompassed early to late adolescence, it is likely that the age effect found is linked to these developmental processes.

The second aim of our study concerned the identification of specific variables related to NSSI and suicidality that could distinguish between the clinical and subclinical groups. As hypothesized, the clinical group showed a higher NSSI frequency than the subclinical group, in line with studies that have considered NSSI frequency as a clinical severity marker [10,11]. In line with our hypothesis, the clinical group also reported the intrapersonal NSSI function “self-punishment” more frequently than the subclinical group. This is also consistent with our previous finding in which the clinical group showed higher severity of the psychoticism dimension, which also includes self-accusation, a factor related to guilt and punishment [72]. Previous literature has highlighted that self-punishment could serve as a way to regulate emotions [17] and is specifically related to guilt [82,83] and self-criticism [84]. Moreover, many studies have already shown that NSSI is associated with self-punishment cognitions [85], self-criticism [85,86], and self-blame [87]. In the clinical group, both self-punishment and the impaired identity dimension were greater than in the subclinical group; thus, further studies are required to investigate the relationship between these variables in young self-harmers. Contrary to our hypothesis, other intrapersonal NSSI functions (i.e., reducing negative emotions, urgency and desire) were not associated with belonging to the clinical group. In our study, both the clinical and subclinical groups showed similar difficulties in identifying and describing emotions; thus, NSSI itself could have the intrinsic property of managing emotions [41,88]. Consistent with the study by Horvath and colleagues (2020) [89], we found that the clinical group reported suicidal ideation more frequently than the subclinical group, even though the percentage of subjects who reported “I think to kill myself but I would not do it” differed slightly between the two groups, indicating that thoughts of death are widespread even in self-harmers without admission to mental health services. In line with this, Guan and colleagues (2012) [90], who enrolled a school-based sample of adolescents, found that NSSI increased the risk of developing suicidal ideation. Our finding further confirms the strong relationship between NSSI and suicidality [19,30,31,33] and the need to implement preventative actions in different populations in order to prevent the transition from passive suicidal thoughts to active suicidal ideation. Finally, we wanted to investigate whether the clinical and subclinical groups differed in NSSI disclosure and found that, unlike the subclinical group, almost all adolescents in the clinical group had previously discussed NSSI with somebody. This result is consistent with the review conducted by Simone and colleagues (2020) [44], who reported a greater percentage of NSSI disclosure in samples with high help-seeking behavior. This is the case of the clinical group in which the participants had already experienced first contact with mental health professionals within the process of seeking medical care. Further studies are necessary to better investigate the temporal relationship between NSSI disclosure and help-seeking behaviors, considered as two independent variables [44].

Taken together, our findings suggest that NSSI could be a signal of intrapersonal emotional difficulties in both clinical and subclinical groups of young self-harmers. In the clinical group, these difficulties seem to be accompanied by severe general distress as well as cognitive, relational, and self-esteem problems, thus making the individual global functioning more impaired. Along with this, higher NSSI frequency, suicidal ideation, and NSSI disclosure emerged in the clinical group, all of which are considered markers of severe distress [11,44,89]. In this context, primary and secondary preventive interventions are urgently required to preserve the emotional and relational abilities of young people as much as possible and prevent the worsening of individual global functioning.

The current study has some limitations. First, the sample size, especially for the clinical and subclinical groups, was small, preventing a homogeneous comparison between different groups. As a consequence, the generalization and interpretation of our findings require caution. Secondly, the use of a questionnaire that has no validity scales (such as the SCL-90) is a limitation of our study; furthermore, self-report questionnaires and different assessment contexts among groups can lead to biases linked to social desirability and the voluntary disclosure of individual aspects. A multi-method and multi-informant perspective is needed to better understand self-harming and the associated factors across different populations. Another limitation is the lack of data collection concerning sociodemographic variables. In fact, from the perspective of developing primary and secondary preventative interventions, further studies that consider multiple kinds of variables (e.g., sociodemographic, family, environmental, and health service utilization variables) are needed to better understand the risk factors for NSSI and mental health service utilization among self-harmers. Our study only included female individuals; hence, our findings may not apply to male individuals. Furthermore, the study design was cross-sectional; thus, we could not identify the temporal relationship between the group to which participants belonged and the investigated variables. Longitudinal studies are required to better outline the trajectories of self-harming and related variables in different kinds of samples, there by pinpointing the risk factors that may predict the transition from nonclinical to clinical conditions (i.e., receiving medical attention).

## 5. Conclusions

In line with the previous literature, the present study highlighted the presence of differences in alexithymic traits and considered symptom dimensions between self-harmers (i.e., clinical and subclinical groups) and individuals without an NSSI history (i.e., control group). Furthermore, some of the symptom dimensions—namely, self-depreciation, anxiety, psychoticism, and pathological interpersonal relationships—were also found to distinguish between self-harmers admitted to mental health outpatient services (i.e., clinical group) and self-harmers who did not (i.e., subclinical group). The clinical and subclinical groups also seemed to differ in terms of some NSSI-related features: the clinical group reported a higher frequency of NSSI acts, suicidal ideation, and NSSI disclosure than the subclinical group. Moreover, the NSSI function “self-punishment” seemed to mainly characterize the clinical group. To the best of our knowledge, this is the first study that, considering an adolescent population, compares self-harmers admitted to mental health services with self-harmers enrolled in a nonclinical context and without admission to mental health services with regard to symptom dimensions, alexithymic traits, suicidality, and NSSI-related variables. Therefore, our results may have significant implications for clinical practice and in primary and secondary prevention concerning NSSI in the adolescent population. The literature has already highlighted that, in recent years, NSSI has increased, especially among adolescents. Hence, it has become necessary to develop treatment and prevention strategies to address it in both clinical and community contexts. With regard to secondary prevention, our results could support clinicians in identifying individual dimensions that could be targets of interventions in the NSSI clinical population; in particular, it seems that this population is characterized by severe general distress and cognitive, relational, and self-esteem problems, which may impair the individual’s global functioning. Finally, based on our findings on the comparison between the NSSI groups and the control group, primary preventive interventions should target, together with the abovementioned variables, emotional intrapersonal abilities.

## Figures and Tables

**Table 1:** Categories of people with whom subjects had discussed NSSI.

Category of People	Subclinical Group N (%)	Clinical Group N (%)
**Friends**	18(66.6)	24(61.5)
**Family**	8(29.6)	19(48.7)
**Teachers**	1(3.7)	3(7.6)
**Health Professionals**	6(22.2)	22(56.4)
**Others**	2(7.4)	4(10.2)

**Table 2:** Univariate results of the MANCOVA.

	Variable	Control Group Adjusted Mean (SD)	Subclinical Group Adjusted Mean (SD)	Clinical Group Adjusted Mean (SD)	F	*p*	Pairwise Comparison ^1^
SCL-90-R	Somatization	0.89 (0.048)	1.19 (0.111)	1.53 (0.105)	12.24	<0.001	clinical > control **
	Interpersonal Sensitivity	1.04 (0.048)	1.41 (0.111)	1.77 (0.104)	21.81	<0.001	clinical > control ** subclinical > control **
	Anxiety	0.89 (0.045)	1.24 (0.103)	1.83 (0.098)	30.30	<.0001	clinical > control ** subclinical > control ** clinical > subclinical **
	Hostility	0.92 (0.053)	1.34 (0.123)	1.31 (0.116)	8.48	<0.001	clinical > control ** subclinical > control **
	Psychoticism	0.57 (0.039)	0.86 (0.088)	1.22 (0.841)	21.81	<0.001	clinical > control ** subclinical > control ** clinical > subclinical **
TAS-20	DIF	18.1 (0.418)	22.3 (0.936)	24.1 (0.814)	27.14	<0.001	clinical > control ** subclinical > control **
	DDF	15.2 (0.352)	18 (0.786)	18.7 (0.683)	13.48	<.0001	clinical > control ** subclinical > control **
CDI	Pathological Mood	4.09 (0.204)	6.59 (0.459)	7.49 (0.419)	37.55	<0.001	clinical > control ** subclinical > control **
	Pathological Self-depreciation	4.67 (0.173)	6.04 (0.391)	8.13 (0.353)	54.01	<0.001	clinical > control ** subclinical > control ** clinical > subclinical **
	Pathological Interpersonal Relationships	5.18 (0.201)	6.09 (0.453)	8.48 (0.410)	23.28	<0.001	clinical > control ** clinical > subclinical **

** *p* < 0.01; SD = standard deviation; SCL-90-R = Symptom Checklist 90–R; TAS-20 = Toronto Alexithymia Scale–20: CDI = Children’s Depression Inventory; DIF = difficulty in identifying feelings; DDF = difficulty in describing feelings; ^1^ pairwise comparisons were conducted with Bonferroni-corrected post hoc tests.

## Data Availability

The data presented in this study are available upon reasonable request to the corresponding author. The data are not publicly available because they report private information about the participants.
